# Adverse childhood experiences, unhealthy lifestyle, and nonsuicidal self-injury: findings from six universities in Shaanxi province, China

**DOI:** 10.3389/fpubh.2023.1199882

**Published:** 2023-06-15

**Authors:** Lei Zhang, Wenhua Wang, Yan Chen, Aisimila Abudoula, Xue Wang, Xiaoxiao Yuan, Yi Luo, Mingyang Wu, Le Ma

**Affiliations:** ^1^School of Public Health, Xi’an Jiaotong University Health Science Center, Xi’an, China; ^2^Shaanxi Medical Association, Xi’an, China; ^3^Shaanxi Provincial Health Industry Association Service Center, Xi’an, China; ^4^Changjun Kaifu Middle School, Changsha, China; ^5^Department of Maternal and Child Health, Xiangya School of Public Health, Central South University, Changsha, China

**Keywords:** nonsuicidal self-injury, adverse childhood experiences, lifestyle, epidemiology, college students

## Abstract

**Background:**

Nonsuicidal self-injury (NSSI) is a serious public health problem. The role of adverse childhood experiences (ACEs) and lifestyle on the risk for NSSI is still underexplored, especially among college students. We aimed to investigate the association of ACEs with the risk of NSSI, and effect modifications by lifestyle among college students.

**Methods:**

A total of 18,723 college students from six universities were recruited through a multistage, random cluster sampling method in Shaanxi province, China. The Adverse Childhood Experiences International Questionnaire was used to assess ACEs for each participant, and the Chinese version of the Ottawa Self-injury Inventory was used to assess the presence or absence of NSSI behaviors. Information about lifestyle was collected by a self-designed questionnaire. The associations of NSSI with ACEs and lifestyle were analyzed using logistic regression models. Furthermore, we constructed a combination score of multiple lifestyles and evaluated whether lifestyle modified the effect of ACEs on the risk of NSSI.

**Results:**

The prevalence of NSSI for the past 1 month, 6 months, and 12 months was 3.8, 5.3, and 6.5%, respectively. 82.6% of participants have reported experiencing at least one type of ACEs, and participants with higher levels of ACEs (≥4) were more likely to have higher odds of developing NSSI during the past 1 month (OR, 4.10; 95%CI, 3.38–4.97), 6 months (OR, 4.76; 95%CI, 4.03–5.62), and 12 months (OR, 5.62; 95%CI, 4.83–6.55), as compared with participants with low levels of ACEs (0–1). There were additive interactions between ACEs and lifestyle. Compared with participants with low levels of ACEs and healthy lifestyle, participants with high levels of ACEs and unhealthy lifestyle had the highest odds of NSSI during the past 1 month (OR, 5.56; 95%CI, 3.80–8.31), 6 months (OR, 6.62; 95%CI, 4.73–9.42), and 12 months (OR, 7.62; 95%CI, 5.59–10.52).

**Conclusion:**

These results suggest that ACEs play an important role in the occurrence of NSSI among college students, especially in those with unhealthy lifestyle. Our findings may help develop targeted intervention strategies for the prevention of NSSI.

## 1. Introduction

Suicide is a serious global public health issue. Globally, more than 700 thousands people die by suicide every year (1), accounting for approximately 1.3% of all deaths. According to previous reports, suicide has been considered to be the second leading cause of death among young individuals aged 15–29 years worldwide (2). Moreover, reports suggest that the incidence of completed suicide among this younger age group in China has been on the rise (3). Given that individuals within this age bracket was still have a long life-cycle, further action, strengthening and acceleration of ongoing efforts in suicide prevention are crucially needed, especially in the special era of accelerate pace of life, and rapid social-economic development in China (4).

Nonsuicidal self-injury (NSSI) is defined as the deliberate direct destruction or alteration of body tissue without a conscious suicidal intent, which was regarded as a gateway of suicide behavior (5, 6). Repeated NSSI will habituate individuals to not fear physical pain, lowering psychological resistance to engage in lethal self-harm, thereby increasing the likelihood of suicide attempts. Therefore, NSSI may consequently amplify an individual’s suicide risk according to the Three-Step Theory. Thus, exploring potential NSSI-related intervenable determinants is essential to reduce the prevalence of NSSI and, ultimately, to reduce suicidal behavior.

Multiple factors were identified as potential causes of NSSI, which involved genetic, biological, psychological, physiological, and other factors (7). Notably, previous clinical observations and cohort studies have emphasized the critical role of early-life adversity in the development and progression of poor mental health conditions, and the adverse childhood experiences (ACEs) were identified as a significant predictor of NSSI (7–9) and also considered to be a “toxic stress.” However, most studies of the relationship between ACEs and NSSI were mainly on the basis of inpatients or adolescents, and the investigation on this issue needs to be strengthened among college students who are facing the development challenge of transitioning to adulthood (10). College students are vulnerable to external stressors during this period (10) due to peer pressure, academic pressure, and social anxiety, especially those with their own vulnerabilities and childhood adversities. In addition, many health-related behaviors (e.g., smoking, alcohol drinking, unhealthy diet, etc.) originate during adolescence and frequently lead to impaired adult health conditions (11, 12). Recent studies have provided convincing evidence that unhealthy lifestyle behaviors may contribute to both psychosocial and physical disorders (13, 14), and such lifestyles may influence each other in a clustered fashion instead of acting independently on one’s health (12). But the extent to which the occurrence of NSSI can be influenced by modifiable lifestyle factors is unknown, regarding multiple behaviors as a lifestyle risk index may provide new insight into the related issue. Moreover, although people with multiple unhealthy lifestyles may be more sensitive to the adverse effects of various forms of toxic exposures (15), it is still unclear whether unhealthy lifestyle is an exacerbating factor on the association between the “toxic stress” -ACEs- and NSSI. Given that unhealthy lifestyle and ACEs were generally related to immune dysregulation (16, 17), the cumulative level of inflammation of the two drivers may have a more profound impact on the adverse health conditions. Therefore, this study hypothesizes that unhealthy lifestyle might be an exacerbating factor on the association of ACEs with NSSI.

Therefore, we conducted this study to address the associations of NSSI with ACEs and lifestyle risk index (a combination of modifiable, health-related behaviors) among college students, and to further explore the joint effect of the lifestyle and ACEs on the risk of NSSI.

## 2. Methods

### 2.1. Study design and participants

Through a multistage, random cluster sampling method, participants were recruited from universities from October to November 2022. Specifically, we randomly selected a total of 6 universities from 57 universities in Shaanxi province, China. Then, we selected approximately 2–4 classes from all the colleges and grades in each sampled university, resulting in a total of 20,165 undergraduates in 559 selected classes were invited to participate in the baseline survey and planned to follow up every semester. Before conducting surveys, each selected class has two class cadres who have received standardized training to guide other classmates to fill out the structured questionnaire using a Quick Response code (QR Code). We also set a calculation question and a choice question in the structured questionnaire for quality control. Finally, a total of 19,622 students submitted their online questionnaires. For the present study, we excluded students who failed to fill the questionnaire completely, completed in a short time (<500 s, determined by a pretest and the 1th percentile calculation), and have invalid questionnaire assessed by logic questions, leaving a total of 18,723 students included for the final analyses. All participants gave their electronic informed consent prior to participate in the study. The study was approved by the Ethics Committee of The Second Affiliated Hospital of Xi’an Jiaotong University (Approval number: 2022–248) and conducted in accordance with the principles of the Declaration of Helsinki.

### 2.2. Measures

#### 2.2.1. Demographic variables

Socio-demographic characteristics including birthdate, gender, grade, registered permanent residence, sibship, parental educational attainment, lifestyle (e.g., smoking, drinking, diet, etc.), height, and weight were collected by a self-designed general information questionnaire.

#### 2.2.2. Adverse childhood experiences

The Adverse Childhood Experiences International Questionnaire (ACE-IQ) developed by the WHO was used to assess ACEs in participants. Briefly, a total of 13 domains of adverse experiences during the first 18 years of their life were asked. There 13 domains include physical abuse, emotional abuse, sexual abuse, family substance use, family incarceration, family mental illness, domestic violence, parental death or separation, emotional neglect, physical neglect, bullying, community violence, and collective violence. Ho et al. translated the ACE-IQ and applied it to the Chinese population, suggesting a good reliability and validity (18). Overall, the Cronbach’s alpha coefficient of the ACE-IQ was measured as 0.69 in the present study. In this study, a response of “ever” for each domain was coded one score, then we summed the total score of the 13 domains. The summary score of 13 domains ranged from 0 to 13, with a higher score denoting more childhood adversities. According to previous studies on the cut-off values of ACEs and the distribution of ACEs in the present study (19, 20), we then classified participants into the following three groups based the ACEs score: low levels of ACEs (0–1), intermediate levels of ACEs (2–3), and high levels of ACEs (≥4).

#### 2.2.3. Nonsuicidal self-injury

The Chinese version of the Ottawa Self-injury Inventory was used to assess the presence or absence of NSSI behaviors. This scale was widely used to evaluate the participants’ frequency of 10 items of NSSI (i.e., hitting, head banging, stabbing, pinching, scratching, biting, burning, and cutting) during the past 1 month, 6 months, and 12 months without suicidal intent. If a participant gives an affirmative answer to whether they exhibited one or more abovementioned self-injury behaviors over the past 1 month, 6 months, or 12 months, he/she was regarded as having NSSI behaviors within a specific period of time (21). Zhang et al. has shown a good reliability and validity in Chinese population (22). Overall, the Cronbach’s alpha coefficient of the Chinese version of the Ottawa Self-injury Inventory was measured as 0.90 in the present study.

#### 2.2.4. Lifestyle

Lifestyle behaviors were collected through a standardized questionnaire. As described in previous studies (23, 24), unhealthy lifestyle factors included current smoking, current alcohol drinking, insufficient physical activity, abnormal weight (obesity or underweight), and unhealthy diet. Current smokers were defined as participants who smoked one or more cigarettes during the past 30 days. Current alcohol drinkers were defined as those who had drunk alcohol at least one glass of wine during the past 30 days. Physical activity was measured by the International Physical Activity Questionnaire Short Form (IPAQ-SF), and physical activity categories include low, moderate, and high based on the standard cut off levels of calculated metabolic equivalents (METs) (25). Those who score ‘high’ on the IPAQ-SF mean that they meet any of the following criteria: (a) ≥3 days of vigorous intensity activity and ≥ 1,500 MET minutes per week; (b) ≥7 days of any combination of walking, moderate or vigorous intensity activities and ≥ 3,000 MET minutes per week. Those who score ‘moderate’ on the IPAQ-SF mean that they meet any of the following criteria: (a) ≥3 days of vigorous intensity activity for at least 20 min per day; (b) ≥5 days of moderate intensity activity and/or walking of at least 30 min per day; (c) ≥5 days of any combination of walking, moderate or vigorous intensity activities and ≥ 600 MET minutes per week. Those with ‘low’ levels of physical activity was participants who did not meet any of the criteria for either ‘moderate’ or ‘high’ levels of physical activity. In this study, the low category of physical activity was considered as unhealthy. Body mass index (BMI) was calculated by dividing body weight by the square of height, while underweight and obesity were defined as BMI less than 18.5 kg/m^2^, and higher than 28 kg/m^2^, respectively. In consistent with previous studies, unhealthy diet was defined as participants who ate red meat every day or vegetables/fruits less than daily (24). Each unhealthy lifestyle was assigned one score, and the summed score of the five behaviors was the lifestyle risk index, which also refers to the term of unhealthy lifestyle score, ranging from 0 to 5 (Supplementary Table S1) (26). According to previous studies on the cut-off values of unhealthy lifestyle score and the distribution of unhealthy lifestyle score in the present study (27, 28), participants were classified into three categories according to their unhealthy lifestyle score (favorable lifestyle, ≤1; intermediate lifestyle, 2; unhealthy lifestyle, ≥3).

#### 2.2.5. Others

We additionally assessed the depressive symptoms using the self-rating depression scale (SDS). The SDS includes 20 items which are scored between 1 (never or very infrequency) and 4 (most or all of the time) and reflects their recent feelings for nearly 1 week. We then converted the summed score of the SDS for each participant to a standard score by multiplying it by 1.25, and the presence of depressive symptoms was defined as a standard score higher than 50 (29). The Pittsburgh Sleep Quality Index (PSQI) including 18 items was used to evaluate the sleep quality of the last month, which yields seven domains including sleep latency, duration of sleep, habitual sleep efficiency, sleeps disturbances, use of sleep medications, daytime dysfunction, and overall sleep quality. The total score of the abovementioned seven domains yields a global PSQI score ranging from 0 to 21, with a higher score (≥8) defined as sleep disorder or poor sleep quality (30, 31). Because previous studies have reported a significant relationship between social support and improved mental health conditions, the Adolescent Social Support Scale (ASSS) including 16 items was used to ascertained social support, with higher scores indicated a higher level of social support. The Cronbach’s alpha coefficients of the SDS, PSQI, and ASSS were measured as 0.88, 0.85, and 0.98, respectively.

### 2.3. Statistical analysis

All data were implemented in R 4.0.2 software.^1^ Continuous and categorical variables were presented as mean ± standard deviation (SD) and counts (percentages), respectively. Descriptive statistics were calculated for demographic variables and compared using chi-squared test for category variables while t-test for continuous variables. Logistic regression models were used to estimate the associations of NSSI with ACEs and lifestyle and to calculate the odds ratios (ORs) and 95% confidence intervals (CIs). We first ran an analysis for the association between total ACEs score and NSSI, with adjustment for gender, grade, race, registered permanent residence, sibship, parental education, lifestyle, sleep quality, depression symptoms, and social support. Then, the ACEs categories were incorporated into models to explore potential nonlinear relationship between ACEs and NSSI. Similarly, we also estimated the relationship between lifestyle and NSSI with logistic regression model. We additionally examined the effects of the combination of ACEs and lifestyle on the risk of NSSI (9 categories with low levels of ACES and healthy lifestyle as reference). The additive interaction was evaluated by using two indexes: the relative excess risk due to the interaction (RERI) and the attributable proportion due the interaction (AP) (32). The formulas of RERI and AP calculation were set as follows: RERI = RERI_11_-RERI_10_-RERI_01_ + 1; AP = RERI/RR_11_. The 95% CIs for the RERI and AP were computed by simulating 5,000 bootstrap samples from the dataset used for estimation (33). If the CIs of the RERI and AP include 0 suggest no significant additive interaction. The main R packages used in this study included ‘epiR’, ‘sjPlot’, ‘ggplot2’, and ‘forestplot’.

## 3. Results

Table 1 lists the baseline characteristics of the study population. A total of 18,723 (males, 34.9%) university students were included in this study. Most participants were Han nationality (97.0%), more than half were rural residents (54.0%), and 29.5% were from single child family. The prevalence of NSSI for the past 1 month, 6 months, and 12 months was 3.8, 5.3, and 6.5%, respectively. Of the 18,723 participants, 82.6% reported experiencing at least one type of ACEs and 9.8% reported experiencing at least four types of ACEs.

**Table 1:** The baseline characteristics of the study population (*N* = 18,723).

Characteristics	Past 1-month NSSI		*p*	Past 6-month NSSI		*p*	Past 12-month NSSI		*p*
	Never	Ever		Never	Ever		Never	Ever	
Gender, *n* (%)			0.509			0.239			0.018
male	6,290 (96.3)	241 (3.7)		6,203 (95)	328 (5)		6,143 (94.1)	388 (5.9)	
female	11,717 (96.1)	475 (3.9)		11,529 (94.6)	663 (5.4)		11,357 (93.2)	835 (6.8)	
Grade, *n* (%)			0.0017			0.25			0.03
1st	5,287 (97)	166 (3)		5,179 (95)	274 (5)		5,057 (92.7)	396 (7.3)	
2nd	4,267 (95.5)	201 (4.5)		4,214 (94.3)	254 (5.7)		4,175 (93.4)	293 (6.6)	
3rd	4,221 (96)	178 (4)		4,153 (94.4)	246 (5.6)		4,120 (93.7)	279 (6.3)	
4th+	4,232 (96.1)	171 (3.9)		4,186 (95.1)	217 (4.9)		4,148 (94.2)	255 (5.8)	
Race, n (%)			0.451			0.23			0.265
Han	17,479 (96.2)	691 (3.8)		17,215 (94.7)	955 (5.3)		16,990 (93.5)	1,180 (6.5)	
others	528 (95.5)	25 (4.5)		517 (93.5)	36 (6.5)		510 (92.2)	43 (7.8)	
Registered permanent residence, n (%)			<0.001			<0.001			<0.001
Rural	9,774 (96.6)	343 (3.4)		9,636 (95.2)	481 (4.8)		9,536 (94.3)	581 (5.7)	
Urban	8,233 (95.7)	373 (4.3)		8,096 (94.1)	510 (5.9)		7,964 (92.5)	642 (7.5)	
Sibship, *n* (%)			0.036			0.288			0.21
Being single child	5,292 (95.7)	237 (4.3)		5,221 (94.4)	308 (5.6)		5,148 (93.1)	381 (6.9)	
Having sibling(s)	12,715 (96.4)	479 (3.6)		12,511 (94.8)	683 (5.2)		12,352 (93.6)	842 (6.4)	
Maternal educational attainment, *n* (%)			0.274			0.5			0.144
Middle school or under	11,597 (96.3)	443 (3.7)		11,417 (94.8)	623 (5.2)		11,282 (93.7)	758 (6.3)	
High school	3,630 (96.1)	148 (3.9)		3,576 (94.7)	202 (5.3)		3,524 (93.3)	254 (6.7)	
College or above	2,780 (95.7)	125 (4.3)		2,739 (94.3)	166 (5.7)		2,694 (92.7)	211 (7.3)	
Paternal educational attainment, *n* (%)			0.257			0.398			0.446
Middle school or under	9,858 (96.3)	374 (3.7)		9,707 (94.9)	525 (5.1)		9,583 (93.7)	649 (6.3)	
High school	3,954 (95.8)	175 (4.2)		3,894 (94.3)	235 (5.7)		3,856 (93.4)	273 (6.6)	
College or above	4,195 (96.2)	167 (3.8)		4,131 (94.7)	231 (5.3)		4,061 (93.1)	301 (6.9)	
Sleep disorder, *n* (%)			<0.001			<0.001			<0.001
No	15,283 (97.6)	375 (2.4)		15,100 (96.4)	558 (3.6)		14,953 (95.5)	705 (4.5)	
Yes	2,724 (88.9)	341 (11.1)		2,632 (85.9)	433 (14.1)		2,547 (83.1)	518 (16.9)	
Depressive symptoms, n (%)			<0.001			<0.001			<0.001
No	16,900 (96.7)	574 (3.3)		16,658 (95.3)	816 (4.7)		16,453 (94.2)	1,021 (5.8)	
Yes	1,107 (88.6)	142 (11.4)		1,074 (86)	175 (14)		1,047 (83.8)	202 (16.2)	
Social support score, mean(SD)	67.4 (15)	59.4 (15.6)	<0.001	67.5 (15)	59.6 (15.2)	<0.001	67.6 (15)	60 (14.9)	<0.001
Healthy lifestyle, *n* (%)			<0.001			<0.001			<0.001
Healthy	3,169 (97.3)	87 (2.7)		3,141 (96.5)	115 (3.5)		3,102 (95.3)	154 (4.7)	
Intermediate	8,385 (96.8)	275 (3.2)		8,267 (95.5)	393 (4.5)		8,168 (94.3)	492 (5.7)	
Unhealthy	6,384 (94.8)	349 (5.2)		6,256 (92.9)	477 (7.1)		6,162 (91.5)	571 (8.5)	
ACEs, median (IQR)	1 (1–2)	2 (1–4)	<0.001	1 (1–2)	2 (1–4)	<0.001	1 (1–2)	2 (1–4)	<0.001
0–1	13,206 (97.9)	281 (2.1)		13,118 (97.3)	369 (2.7)		13,044 (96.7)	443 (3.3)	
2–3	3,045 (94.3)	183 (5.7)		2,950 (91.4)	278 (8.6)		2,889 (89.5)	339 (10.5)	
≥4	1756 (87.5)	252 (12.5)		1,664 (82.9)	344 (17.1)		1,567 (78)	441 (22)	

NSSI-1 m, NSSI during the past 1 month; NSSI-6 m, NSSI during the past 6 months; NSSI-12 m, NSSI during the past 12 months; SD, standard deviation; IQR, inter-quartile range; ACEs, adverse childhood experiences.

**Figure 1:**
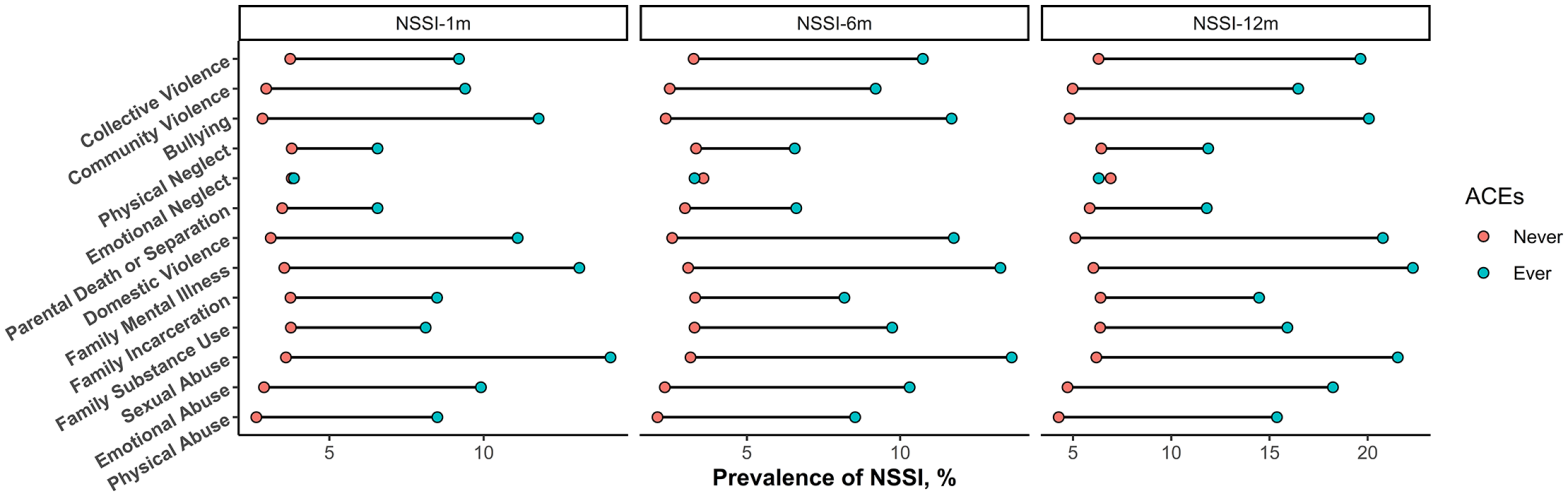
The prevalence of NSSI across different ACEs. Note: NSSI, Nonsuicidal self-injury; ACEs, adverse childhood experiences;

Students experienced various types of ACEs had higher prevalence of NSSI during the past 1 month, 6 months, or 12 months (Figure 1 and Supplementary Table S2). As depicted in Table 2, we summed the cumulated ACEs score as an exposure variable and analyzed their association with NSSI. After adjustment for gender, grade, race, registered permanent residence, sibship, parental education, healthy lifestyle, sleep quality, depression symptoms, and social support, each unit increases in ACEs score was significantly associated with the higher odds of NSSI during the past 1 month (OR, 1.23; 95%CI, 1.19–1.27), 6 months (OR, 1.26; 95%CI,1.23–1.30), and 12 months (OR, 1.30; 95%CI, 1.26–1.33). We then classified participants into three groups according to the ACEs score, to explore potential nonlinearity correlations; and results showed that participants with higher levels of ACEs (≥4) were more likely to have higher odds of developing NSSI during the past 1 month (OR, 4.10; 95%CI, 3.38–4.97), 6 months (OR, 4.76; 95%CI, 4.03–5.62), and 12 months (OR, 5.62; 95%CI, 4.83–6.55), as compared with participants with low levels of ACEs (0–1).

**Table 2:** Associations between ACEs and NSSI.

ACEs	OR (95%CI)		
	NSSI-1 m	NSSI-6 m	NSSI-12 m
Each unit increases	1.23 (1.19–1.27)	1.26 (1.23–1.30)	1.30 (1.26–1.33)
Low levels (0–1)	Reference	Reference	Reference
Intermediate levels (2–3)	2.23 (1.83–2.72)	2.75 (2.33–3.24)	2.89 (2.49–3.37)
High levels (≥4)	4.10 (3.38–4.97)	4.76 (4.03–5.62)	5.62 (4.83–6.55)

NSSI, Nonsuicidal self-injury; ACEs, adverse childhood experiences; OR, odds ratio; CI, confidence interval; All models adjusted for gender, grade, race, registered permanent residence, sibship, parental education, lifestyle, sleep quality, depression symptoms, and social support.

**Figure 2:**
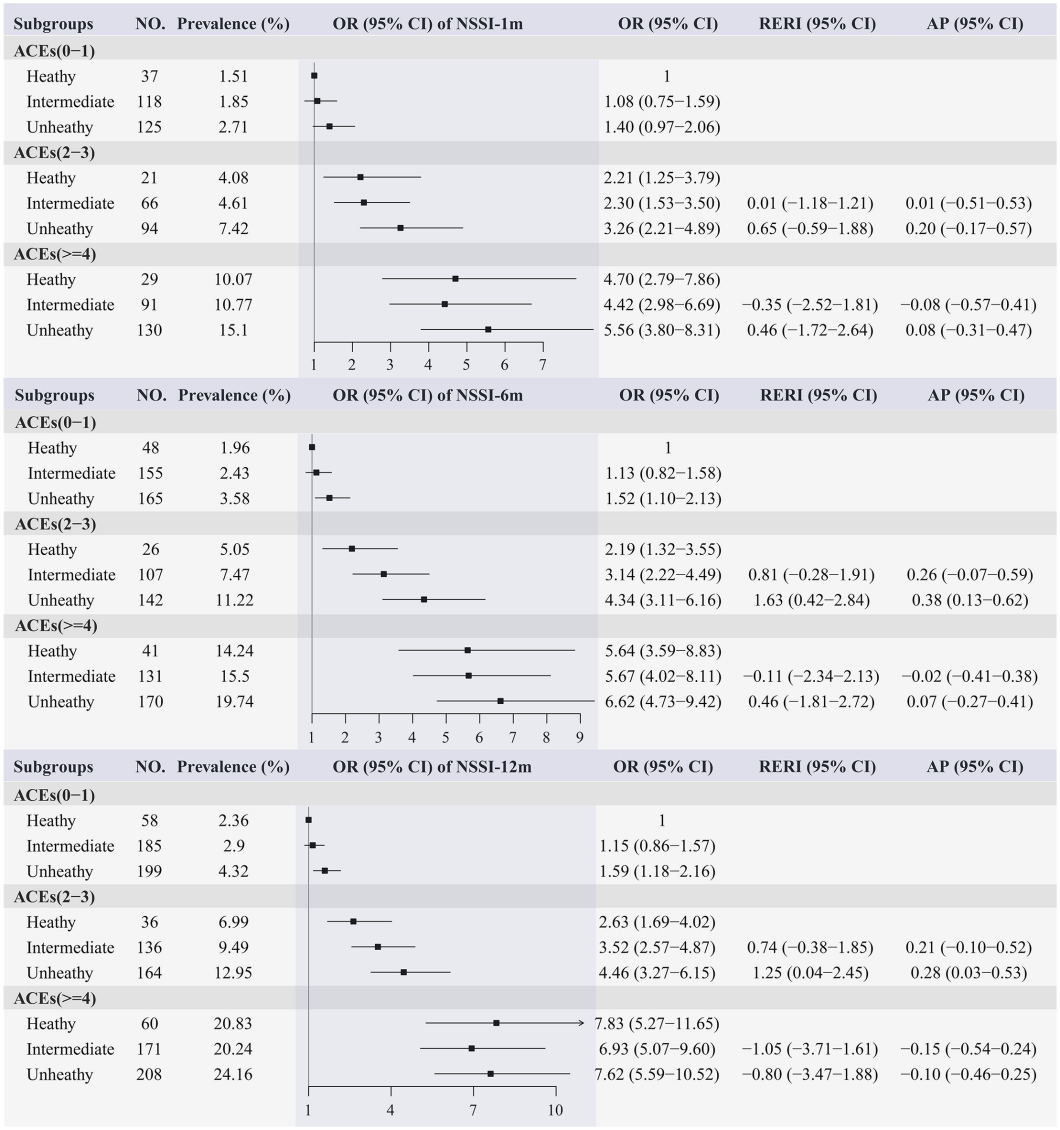
Joint effect of ACEs and lifestyle on the prevalence of NSSI. NSSI, Nonsuicidal self-injury; ACEs, adverse childhood experiences; OR, odds ratio; CI, confidence interval; RERI, relative excess risk due to the interaction; AP, attributable proportion due the interaction; All models adjusted for gender, grade, race, registered permanent residence, sibship, parents’ education, sleep quality, depression symptoms, and social support.

According to Supplementary Table S3 and Table 3, logistic regression revealed that there were significant relationships between lifestyle categories and NSSI. Specifically, compare with participants with a healthy lifestyle, those with an unhealthy lifestyle had significant increased prevalence of NSSI during the past 1 month (OR, 1.34; 95%CI, 1.05–1.73), 6 months (OR, 1.50; 95%CI, 1.21–1.87), and 12 months (OR, 1.38; 95%CI, 1.14–1.68). In addition, we observed a joint effect of unhealthy lifestyle and ACEs on the odds of NSSI that behaved in a dose–response manner; that overall odds of NSSI increased as both ACEs score and unhealthy lifestyle score increased (Figure 2). More specifically, in the multivariable-adjusted model, compared participants with low levels of ACEs and healthy lifestyle, participants with high levels of ACEs and unhealthy lifestyle had the highest odds of NSSI during the past 1 month (OR, 5.56; 95%CI, 3.80–8.31), 6 months (OR, 6.62; 95%CI, 4.73–9.42), and 12 months (OR, 7.62; 95%CI, 5.59–10.52). Additionally, we also observed positive additive interactions of ACEs with unhealthy lifestyle on the prevalence of NSSI (Figures 2 and Supplementary Figure S1). For instance, for intermediate ACEs with an unhealthy lifestyle, the RERI was 1.25 (95% CI, 0.04–2.45) for NSSI during the past 12 months, which suggested that there would be a 1.25 relative excess risk because of the additive interaction, accounting for 28% (3–53%) of the odds of NSSI in participants exposed to both intermediate ACEs and unhealthy lifestyle. Because the unbalanced sex ratio in the present study, we additionally performed a weighted model and results did not change substantially (Supplementary Table S4).

**Table 3:** Associations between unhealthy lifestyle score and NSSI.

Lifestyle	OR (95%CI)		
	NSSI-1 m	NSSI-6 m	NSSI-12 m
Healthy	Reference	Reference	Reference
Intermediate	1.01 (0.79–1.31)	1.15 (0.92–1.43)	1.10 (0.91–1.33)
Unhealthy	1.34 (1.05–1.73)	1.50 (1.21–1.87)	1.38 (1.14–1.68)

NSSI, Nonsuicidal self-injury; OR, odds ratio; CI, confidence interval; All models adjusted for gender, grade, race, registered permanent residence, sibship, parents’ education, ACEs, sleep quality, depression symptoms, and social support.

## 4. Discussion

In the present large-scaled school-based survey, we showed evidence that ACEs and unhealthy lifestyle significantly increased the odds of engaging in NSSI among college students. Furthermore, when examining the joint effects of lifestyle and ACEs, we found that the greatest relative increase in odds of NSSI was observed among those with high levels of ACEs and unhealthy lifestyle. The present study also provides quantitative data about the effect of the additive interaction between lifestyle and ACEs on NSSI.

There is considerable variation in the NSSI prevalence rates across different countries. A review of the prevalence of NSSI in youth per each low- and middle-income country identified in the reports shows 12-month prevalence rates ranging from 15.5 to 31.3% (34). In China, previous studies have indicated a relatively high prevalence or incidence of NSSI among youths. But most of these studies were conducted among middle-school students, while studies on the prevalence of NSSI among college students were still scarce, although this group are facing the development challenge of transitioning to adulthood and are vulnerable to external stressors during this period. In the present study consisted of six universities in Shaanxi province, China, we found that the prevalence of NSSI among college students was 3.8, 5.3, and 6.5% during the past 1 month, 6 months, and 12 months, respectively, which were comparable to the 5.5% previous reported in adults (35). However, it is lower than the prevalence reported in medical students in China. For example, Wan et al. conducted a study among 4,063 medical students in Anhui province, China, and showed a 13.4% prevalence of NSSI during the past 12 months (36). Wu et al. also presented a 9.6% prevalence of NSSI during the past 12 months in medical college students in China (37). The difference in the prevalence of NSSI reported by different studies may be related to study population, assessment tools, and locations. The present study population was sampled from students of different majors in six universities, which may overcome the limitation of the representation of selected population in previous studies conducted only in single university or only among participants of a specific major. Because the related study is still limited so far, further studies are needed in a nationally representative population.

There are increasing interests in studying the association between childhood adversities and NSSI. As shown by previous studies, childhood adversities destroy an individual’s ability to appropriately regulate or manage negative emotional states, resulting in increased risk of emotional problems and extreme distress for individuals (38), and as a result, individuals may be more likely to engage in self-harm behaviors, such as NSSI. Although the association between ACEs and NSSI have been extensively studied among adolescents, investigations on this issue among college students are still limited. To the best of our knowledge, only two studies were conducted to reveal the relationship between ACEs and NSSI among college students. Siobhan O’Neill et al. conducted a small sample sized study in UK (N = 739), and their results indicated a significant relationship between high levels of ACEs and NSSI (39). Chen et al. showed that individuals with high levels of ACEs had significantly higher prevalence of NSSI among Chinese college students (N = 1,036) (40). The present study found significant accumulated effects of the number of ACEs on the odds of NSSI in the past 1 month, 6 months, and 12 months, extending the limited available evidence on the association between childhood adversities and NSSI.

The causes of NSSI behavior in college students are complicated. As mentioned in a recent review (7), problem behaviors (e.g., smoking and drinking) may play important roles in the development of NSSI. Although most of problem behaviors are modifiable and related to lifestyle, no clear modifiable factors have been identified for the link between ACEs and NSSI to date. Recent studies have showed the maintenance or interventions of healthy lifestyle may be related to decreased risks of long-term physical and mental health conditions (41). A more recent study has provided convincing evidence that unhealthy lifestyle trajectory is associated with more than two-fold elevated odds for multiple domains of psychopathological outcomes over 5 years (42). However, studies on the modification effects of lifestyle on the association between ACEs and NSSI are still scarce. One of the reasons may be that most studies lacked statistical power to perform interaction tests or lacked information on potential lifestyles. For the present large-scaled population-based study, we collected detailed information on lifestyles that helps to explore possible interactions. We observed a joint effect of unhealthy lifestyle and ACEs on the odds of NSSI that behaved in a dose–response manner, and the additive interactions of intermediate levels of ACEs with unhealthy lifestyle on the prevalence of NSSI were statistically significant, which implies that individuals adopting an unhealthy lifestyle in daily life may amplify the adverse effect of ACEs on NSSI. To the best of our knowledge, our study is the first to examine the interactions between ACEs and lifestyle on the prevalence of NSSI among college students. The biologic mechanisms underlying the interaction effects of lifestyle on the relationship between ACEs and NSSI remain unclear. We speculate that college students who have been exposed childhood adversities may be particularly sensitive to negative social components of unhealthy lifestyle. Additionally, a recent study has reported a link between increased inflammatory levels and the development of NSSI (43). Given that unhealthy lifestyle and childhood adversities were generally related to immune dysregulation (16, 17), we speculate that the accumulated immune dysregulation caused by the combination of ACEs and unhealthy lifestyle may be another plausible mechanism behind the observed interactions. However, more detailed specific mechanisms are needed to be further clarified in future studies.

Findings from the present study have important practical implications. Since these lifestyles are modifiable and adopting a healthy lifestyle is beneficial to individuals’ physical health and mental well-being, from an individual’s perspective, college students may benefit from healthy lifestyle to combat the hazards of childhood adversities, so as to reduce the risk of NSSI. Our findings may also be helpful to formulate targeted intervention strategies to reduce the adverse effect of ACEs on NSSI. For instance, if lifestyle-specific interventions (e.g., stop smoking, promote healthy diet, or increase physical activity) were conducted in college students, researchers are needed to further consider the adverse effects of childhood adversities and the potential interactions between childhood adversities and lifestyle. Notably, as revealed by a recent randomized controlled trial in Netherland, participants with a history of childhood adversity modified the effect of a lifestyle intervention on women’s body composition (41), which might indirectly confirm the present findings. However, given the cross-sectional study design of the present study and the focus of the abovementioned mentioned study on physiological health, further cohort studies or trials in the field of neuropsychology are needed to illustrate the modification effect of lifestyle.

Some limitations of this study should be noted. First, although most available individual/parental demographic and lifestyle variables were incorporated into the model for adjustment, some residual or unmeasured confounding parameters could have affected the results. For instance, the confounding effect of the timing of childhood adversities may vary depending on sensitive period (44). Second, a self-report retrospective online questionnaire was used to access the prevalence of NSSI, which may result in recall bias. Third, the causal inference was still limited by cross-sectional study design for the present study. Last, the unbalanced sex ratio may limit the extrapolation of this study.

## 5. Conclusion

In conclusion, this study provides evidence suggesting that ACEs and lifestyle play an important role in the occurrence of NSSI among college students. Such findings may help develop targeted intervention strategies for the prevention of NSSI.

## Data availability statement

The original contributions presented in the study are included in the article/Supplementary material, further inquiries can be directed to the corresponding authors.

## Ethics statement

This study was approved by the Ethics Committee of The Second Affiliated Hospital of Xi’an Jiaotong University (Approval number: 2022-248). All participants gave written informed consent before enrolment in the study, which was conducted in accordance with the principles of the Declaration of Helsinki.

## Author contributions

LZ contributed to the conception or design of the paper and drafted the manuscript. LZ, WW, YC, AA, XW, XY, YL, MW, and LM contributed to the acquisition, analysis, or interpretation of data for the work. LZ, MW, and LM provided a critical review of the manuscript. All authors contributed to the article and approved the submitted version.

## Funding

This research was funded by the Medical Research Foundation of Shaanxi Medical Association (MRFSMA2022001), the Natural Science Foundation of Changsha (kq2208302), and the Open Project of Key Laboratory of Environment and Health, Ministry of Education (2022GWKFJJ02).

## Conflict of interest

The authors declare that the research was conducted in the absence of any commercial or financial relationships that could be construed as a potential conflict of interest.

## Supplementary material

The Supplementary material for this article can be found online at: https://www.frontiersin.org/articles/10.3389/fpubh.2023.1199882/full#supplementary-material

## Footnotes

^1^ https://www.r-project.org/
