# Supplementary material for: Adverse childhood experiences, unhealthy lifestyle, and nonsuicidal self-injury: findings from six universities in Shaanxi province, China
Source: Front Public Health. 2023 Jun 15;11:1199882. doi: 10.3389/fpubh.2023.1199882 (PMC10308309; doi:10.3389/fpubh.2023.1199882)
Supplement: Supplementary file 1 [file Table_1.docx]

**Adverse childhood experiences, unhealthy lifestyles, and nonsuicidal self-injury: findings from six universities in Shaanxi province, China**

Lei Zhang ^1,2,3^, Wenhua Wang ^3^, Yan Chen ^4^, Aisimila Abudoula ^5^, Xue Wang ^2^, Xiaoxiao Yuan ^3^, Yi Luo ^3^, Mingyang Wu ^5, *^, Le Ma ^6, *^

^1^ School of Public Health, Xi’an Jiaotong University Health Science Center, Xi’an, China, 710061;

^2^ Shaanxi Medical Association, Xi’an, China, 710003;

^3^ Shaanxi Provincial Health Industry Association Service Center, Xian, China, 710003;

^4^ Changjun Kaifu Middle School, Changsha, China, 410000;

^5^ Department of Maternal and Child Health, Xiangya School of Public Health, Central South University, Changsha, China, 410078;

^6^ School of Public Health, Xi’an Jiaotong University Health Science Center, Xi’an 710061, China;

**Supplementary materials:**

**Table S1 The detailed definitions of unhealthy lifestyle factors**

**Table S2 The associations of ACEs with NSSI**

**Note**: NSSI, Nonsuicidal self-injury; ACEs, adverse childhood experiences; OR, odds ratio; CI, confidence interval; All models adjusted for gender, grade, race, registered permanent residence, sibship, parental education, lifestyle, sleep quality, depression symptoms, and social support.

**Table S3 The associations of lifestyles with NSSI**

**Note**: NSSI, Nonsuicidal self-injury; OR, odds ratio; CI, confidence interval; All models adjusted for gender, grade, race, registered permanent residence, ACEs score, sibship, parental education, sleep quality, depression symptoms, and social support.

**Table S4 Joint effect of ACEs and lifestyle on the prevalence of NSSI in weighted model**

**Note**: NSSI, Nonsuicidal self-injury; ACEs, adverse childhood experiences; OR, odds ratio; CI, confidence interval; All models adjusted for gender, grade, race, registered permanent residence, sibship, parental education, sleep quality, depression symptoms, and social support.

**Table S5 Joint effect of ACEs and lifestyle on the prevalence of NSSI, stratified by gender**

**Note**: NSSI, Nonsuicidal self-injury; ACEs, adverse childhood experiences; OR, odds ratio; CI, confidence interval; All models adjusted for grade, race, registered permanent residence, sibship, parental education, sleep quality, depression symptoms, and social support.

**Figure S1 Effect of lifestyle on the association between ACEs and NSSI.**

**Note**: NSSI, Nonsuicidal self-injury; ACEs, adverse childhood experiences; All models adjusted gender, grade, race, registered permanent residence, sibship, parents’ education, sleep quality, depression symptoms, and social support.

**Table S1 The detailed definitions of unhealthy lifestyle factors**

| **Unhealthy lifestyle factors** | **Definition** |
| --- | --- |
| Smoking | current smoking |
| Drinking | current drinking |
| Diet | one or more of the following 3 characteristics, as assessed by food frequency questionnaire 1.Fruits: <7times/week 2.Vegetables: <7times/week 3.Red meat: ≥7times/week |
| Physical activity | IPAQ questionnaire to define the low activity category |
| Abnormal weight | BMI≥28 kg/m^2^ or BMI<18.5 kg/m^2^ |

**Table S2 The associations of ACEs with NSSI**

| ACEs types | Categories | OR (95%CI) | | |
| --- | --- | --- | --- | --- |
|  |  | NSSI-1m | NSSI-6m | NSSI-12m |
| Physical Abuse | never | Ref (1) | Ref (1) | Ref (1) |
|  | ever | **2.50 (2.13-2.93)** | **2.90 (2.53-3.32)** | **3.17 (2.80-3.59)** |
| Emotional Abuse | never | Ref (1) | Ref (1) | Ref (1) |
|  | ever | **2.40 (2.02-2.84)** | **2.77 (2.39-3.20)** | **3.14 (2.75-3.58)** |
| Sexual Abuse | never | Ref (1) | Ref (1) | Ref (1) |
|  | ever | **2.66 (1.95-3.59)** | **2.61 (1.97-3.43)** | **2.68 (2.06-3.45)** |
| Family Substance Use | never | Ref (1) | Ref (1) | Ref (1) |
|  | ever | 1.33 (0.84-2.02) | **1.51 (1.02-2.16)** | **1.81 (1.29-2.49)** |
| Family Incarceration | never | Ref (1) | Ref (1) | Ref (1) |
|  | ever | **1.60 (1.03-2.40)** | **1.74 (1.19-2.47)** | **1.78 (1.26-2.47)** |
| Family Mental Illness | never | Ref (1) | Ref (1) | Ref (1) |
|  | ever | **2.56 (1.93-3.34)** | **2.71 (2.12-3.44)** | **3.00 (2.39-3.74)** |
| Domestic Violence | never | Ref (1) | Ref (1) | Ref (1) |
|  | ever | **2.55 (2.11-3.07)** | **2.95 (2.51-3.46)** | **3.48 (3.01-4.02)** |
| Parental Death or Separation | never | Ref (1) | Ref (1) | Ref (1) |
|  | ever | **1.58 (1.29-1.93)** | **1.76 (1.49-2.09)** | **1.81 (1.54-2.10)** |
| Emotional Neglect | never | Ref (1) | Ref (1) | Ref (1) |
|  | ever | **1.25 (1.06-1.47)** | **1.36 (1.18-1.57)** | **1.38 (1.21-1.56)** |
| Physical Neglect | never | Ref (1) | Ref (1) | Ref (1) |
|  | ever | 1.28 (0.78-1.99) | 1.38 (0.91-2.02) | **1.45 (1.00-2.06)** |
| Bullying | never | Ref (1) | Ref (1) | Ref (1) |
|  | ever | **3.11 (2.61-3.69)** | **3.46 (2.98-4.01)** | **3.65 (3.18-4.18)** |
| Community Violence | never | Ref (1) | Ref (1) | Ref (1) |
|  | ever | **2.53 (2.13-3.00)** | **2.76 (2.38-3.20)** | **3.02 (2.63-3.45)** |
| Collective Violence | never | Ref (1) | Ref (1) | Ref (1) |
|  | ever | **1.73 (1.13-2.55)** | **2.26 (1.61-3.12)** | **2.70 (1.99-3.61)** |

**Note**: NSSI, Nonsuicidal self-injury; ACEs, adverse childhood experiences; OR, odds ratio; CI, confidence interval;

All models adjusted for gender, grade, race, registered permanent residence, sibship, parental education, lifestyle, sleep quality, depression symptoms, and social support.

**Table S3 The associations of lifestyles with NSSI**

| Lifestyles | OR (95%CI) | | |
| --- | --- | --- | --- |
|  | NSSI-1m | NSSI-6m | NSSI-12m |
| Current smoking | 1.38 (1.11-1.70) | 1.52 (1.26-1.82) | 1.56 (1.32-1.85) |
| Current drinking | 1.99 (1.68-2.36) | 2.00 (1.73-2.32) | 1.97 (1.72-2.25) |
| Low physical activity | 0.83 (0.62-1.14) | 0.87 (0.67-1.14) | 1.01 (0.79-1.31) |
| Unhealthy diet | 0.95 (0.80-1.15) | 0.94 (0.81-1.10) | 0.90 (0.78-1.04) |
| Unhealthy weight | 0.98 (0.83-1.16) | 1.06 (0.92-1.23) | 0.97 (0.85-1.11) |

**Note**: NSSI, Nonsuicidal self-injury; OR, odds ratio; CI, confidence interval; All models adjusted for gender, grade, race, registered permanent residence, ACEs score, sibship, parental education, sleep quality, depression symptoms, and social support.

**Table S4 Joint effect of ACEs and lifestyle on the prevalence of NSSI in weighted model**

| Subgroups | OR (95%CI) | | |
| --- | --- | --- | --- |
|  | NSSI-1m | NSSI-6m | NSSI-12m |
| ACEs (0-1) |  |  |  |
| Heathy | Ref (1) | Ref (1) | Ref (1) |
| Intermediate | 1.14 (0.78-1.71) | 1.12 (0.81-1.59) | 1.12 (0.83-1.54) |
| Unheathy | 1.49 (1.03-2.23) | 1.55 (1.12-2.19) | 1.61 (1.19-2.20) |
| ACEs (2-3) |  |  |  |
| Heathy | 2.52 (1.43-4.34) | 2.39 (1.45-3.86) | 2.67 (1.71-4.12) |
| Intermediate | 2.46 (1.62-3.81) | 3.18 (2.23-4.59) | 3.51 (2.54-4.89) |
| Unheathy | 3.37 (2.26-5.13) | 4.30 (3.06-6.14) | 4.47 (3.27-6.20) |
| ACEs (>=4) |  |  |  |
| Heathy | 4.86 (2.85-8.24) | 5.77 (3.66-9.08) | 7.83 (5.24-11.72) |
| Intermediate | 4.73 (3.15-7.26) | 5.67 (3.99-8.19) | 6.74 (4.89-9.41) |
| Unheathy | 6.13 (4.16-9.28) | 6.98 (4.97-9.99) | 7.80 (5.71-10.82) |

**Note**: NSSI, Nonsuicidal self-injury; ACEs, adverse childhood experiences; OR, odds ratio; CI, confidence interval; All models adjusted for gender, grade, race, registered permanent residence, sibship, parental education, sleep quality, depression symptoms, and social support.

**Table S5 Joint effect of ACEs and lifestyle on the prevalence of NSSI, stratified by gender**

| Gender | Subgroups | OR (95%CI) | | |
| --- | --- | --- | --- | --- |
|  |  | Past 1-month NSSI | Past 6-month NSSI | Past 12-month NSSI |
| Male | ACEs(0-1) |  |  |  |
|  | Heathy | Ref (1) | Ref (1) | Ref (1) |
|  | Intermediate | 1.08 (0.75-1.59) | 1.13 (0.82-1.58) | 1.15 (0.86-1.57) |
|  | Unheathy | 1.40 (0.97-2.06) | 1.52 (1.10-2.13) | 1.59 (1.18-2.16) |
|  | ACEs(2-3) |  |  |  |
|  | Heathy | 2.21 (1.25-3.79) | 2.19 (1.32-3.55) | 2.63 (1.69-4.02) |
|  | Intermediate | 2.30 (1.53-3.50) | 3.14 (2.22-4.49) | 3.52 (2.57-4.87) |
|  | Unheathy | 3.26 (2.21-4.89) | 4.34 (3.11-6.16) | 4.46 (3.27-6.15) |
|  | ACEs(>=4) |  |  |  |
|  | Heathy | 4.70 (2.79-7.86) | 5.64 (3.59-8.83) | 7.83 (5.27-11.65) |
|  | Intermediate | 4.42 (2.98-6.69) | 5.67 (4.02-8.11) | 6.93 (5.07-9.60) |
|  | Unheathy | 5.56 (3.80-8.31) | 6.62 (4.73-9.42) | 7.62 (5.59-10.52) |
| Female | ACEs(0-1) |  |  |  |
|  | Heathy | Ref (1) | Ref (1) | Ref (1) |
|  | Intermediate | 0.96 (0.63-1.50) | 1.14 (0.78-1.70) | 1.21 (0.86-1.74) |
|  | Unheathy | 1.23 (0.81-1.94) | 1.45 (0.99-2.17) | 1.54 (1.09-2.23) |
|  | ACEs(2-3) |  |  |  |
|  | Heathy | 1.66 (0.80-3.23) | 1.80 (0.94-3.30) | 2.54 (1.49-4.25) |
|  | Intermediate | 1.99 (1.24-3.24) | 3.03 (2.02-4.64) | 3.52 (2.43-5.18) |
|  | Unheathy | 3.09 (1.96-4.97) | 4.46 (2.98-6.79) | 4.41 (3.03-6.50) |
|  | ACEs(>=4) |  |  |  |
|  | Heathy | 4.50 (2.41-8.25) | 5.40 (3.11-9.27) | 7.82 (4.85-12.63) |
|  | Intermediate | 3.85 (2.43-6.22) | 5.63 (3.75-8.61) | 7.31 (5.06-10.75) |
|  | Unheathy | 4.43 (2.82-7.12) | 5.74 (3.81-8.80) | 7.13 (4.91-10.54) |

**Note**: NSSI, Nonsuicidal self-injury; ACEs, adverse childhood experiences; OR, odds ratio; CI, confidence interval; All models adjusted for grade, race, registered permanent residence, sibship, parental education, sleep quality, depression symptoms, and social support.


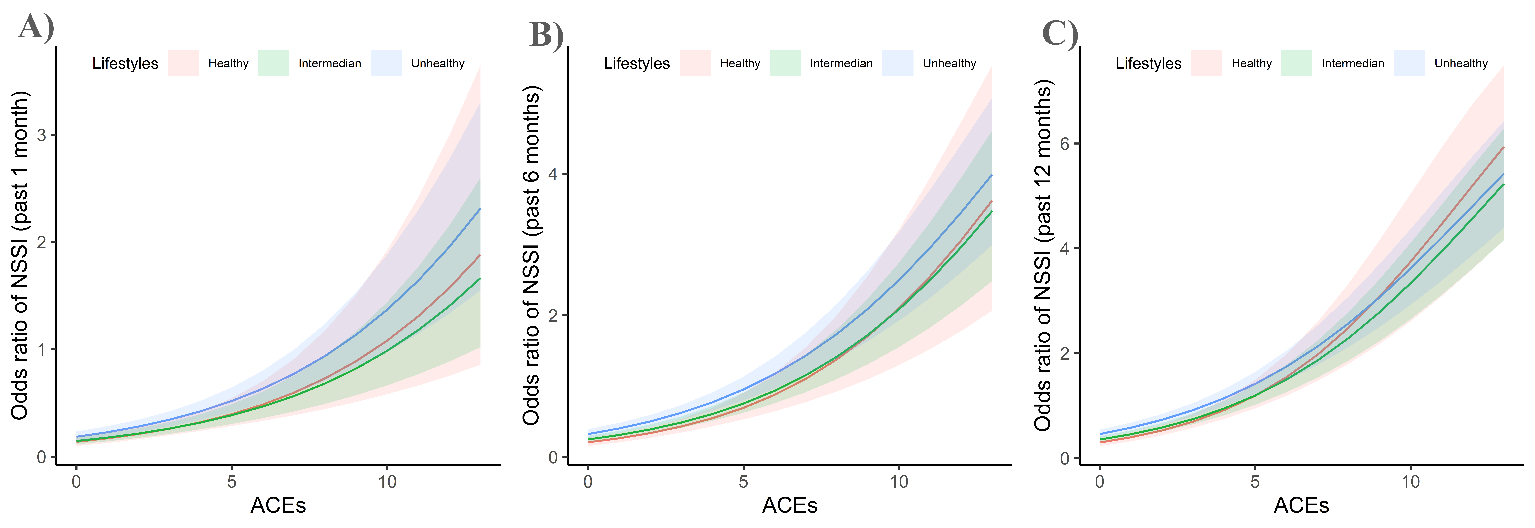


**Figure S1 Effect of lifestyle on the association between ACEs and NSSI.**

**Note**: NSSI, Nonsuicidal self-injury; ACEs, adverse childhood experiences; All models adjusted gender, grade, race, registered permanent residence, sibship, parents’ education, sleep quality, depression symptoms, and social support.
